# Long-term evacuation and obesity: a 12-year follow-up comparative study of residents inside and outside Katsurao Village after the Fukushima nuclear disaster

**DOI:** 10.3389/fpubh.2024.1394308

**Published:** 2024-07-02

**Authors:** Naomi Ito, Morihito Takita, Nobuaki Moriyama, Isamu Amir, Ayako Furuyama, Hiroaki Saito, Toshiki Abe, Chika Yamamoto, Mika Sato, Tianchen Zhao, Masaharu Tsubokura

**Affiliations:** ^1^Department of Radiation Health Management, Fukushima Medical University School of Medicine, Fukushima, Japan; ^2^Research Division, Medical Governance Research Institute, Tokyo, Japan; ^3^Department of Public Health, Fukushima Medical University School of Medicine, Fukushima, Japan; ^4^Health Promotion Center, Fukushima Medical University, Fukushima, Japan; ^5^Department of Health Nursing of International Radiation Exposure, Fukushima Medical University School of Medicine, Fukushima, Japan

**Keywords:** Fukushima nuclear accident, aging in place, evacuees, long-term relocation, obesity, long-term care

## Abstract

**Objective:**

Evacuation, owing to a disaster, impacts various aspects of an individual's life, including health status. This study aimed to determine the prevalence of obesity among residents of Katsurao Village, Fukushima Prefecture, after the evacuation order due to the Fukushima nuclear disaster in 2011 was lifted in 2016 and to compare the prevalence of obesity by place of residence (inside or outside the village).

**Methods:**

The number of examinees, sex, age, place of residence, body mass index (BMI), exercise habits, smoking habits, drinking habits, and dietary status were extracted from the results of health checkups since 2016. We compared the BMI of the indigenes of Katsurao Village by place of residence (inside or outside the village) over time.

**Results:**

Although 7 years have passed since 2016, ~70% of the registered residents of Katsurao Village still live outside the village. The obesity rates have consistently been higher among people living outside the village compared to those inside, and the place of residence was the only factor significantly associated with obesity.

**Conclusion:**

The findings of this study suggest early intervention is necessary to prevent health risks associated with disaster evacuation if the evacuation period is prolonged.

## 1 Introduction

Generally, individuals prefer to continue living and aging in a familiar region (1, 2). The idea of “aging in place” represents a central concept of community living (3, 4). Aging in place gives older adults a sense of identity through human relationships, roles, and independent living (5). However, factors such as proximity to disaster zones affect aging in place. Taking measures to facilitate aging in place following such events is an important public health concern.

In Japan, natural disasters occur frequently, and after each disaster, evacuees emerge. A large majority of evacuees settled down in their new residences; however, after the Fukushima Daiichi nuclear power plant accident that occurred in 2011, evacuated residents experienced major changes in their lifestyle and social environment. Currently, more than 30,000 people who evacuated a radioactive atmosphere are still living in temporary housing. Consequently, the effects of such evacuation on the health of residents are multifaceted and long-term (6). For example, evacuees experience severe levels of depression (7), and the number of evacuees that require nursing care services increases (8, 9). Furthermore, worsening health indicators, including an increase in hypertension and diabetes as well as a sharp increase in the number of individuals with obesity (10, 11), have been observed. However, since the evacuation order has been lifted, only a few studies have examined aging in place, specifically the differences in the health status between residents who returned to their original locations and those who did not, which remains insufficiently evaluated.

**Figure 1:**
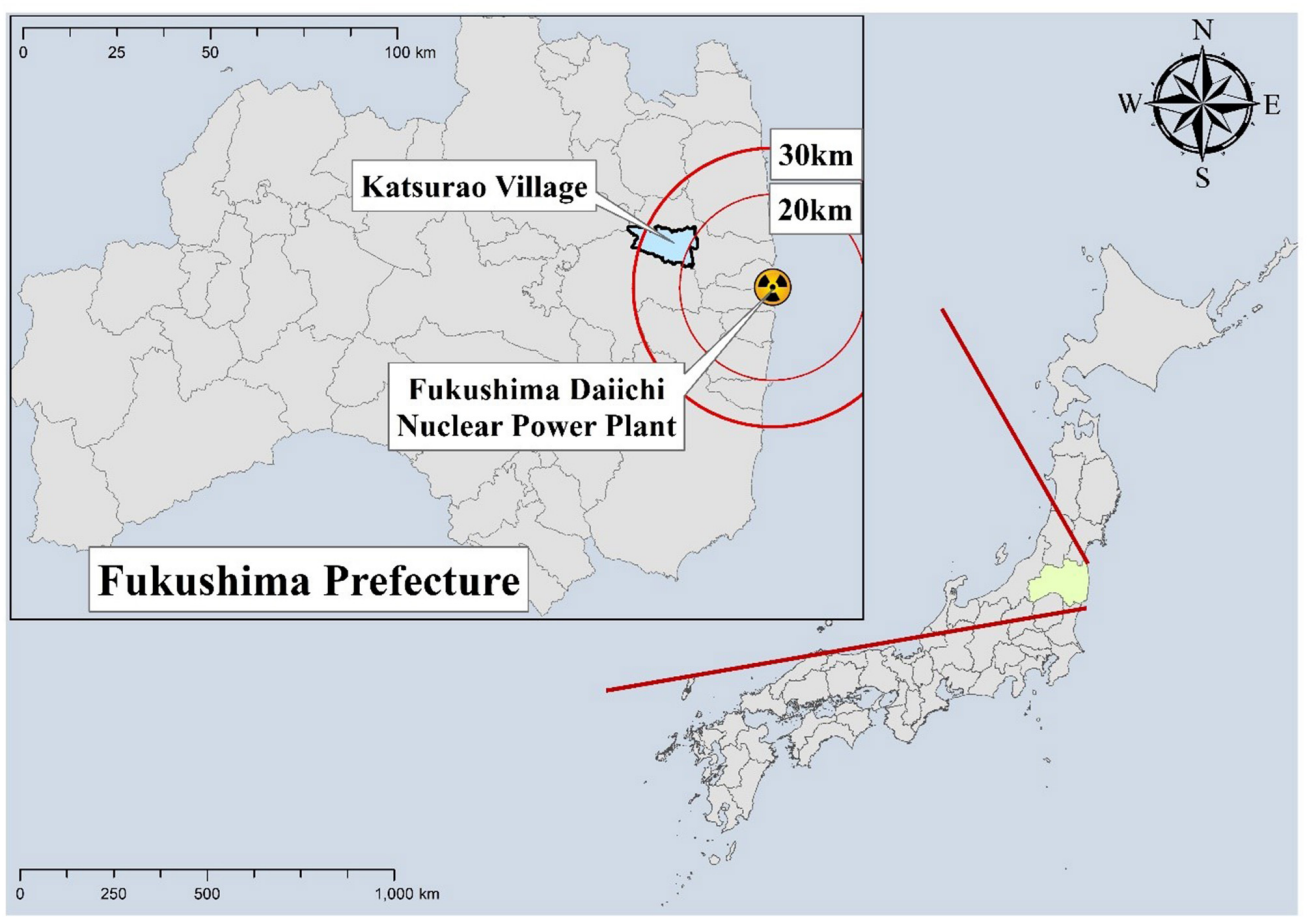
Location of Katsurao Village in relation to the Fukushima Daiichi Nuclear Power Plant.

Katsurao Village is located within a 20–30 km radius of the Fukushima Daiichi nuclear power plant (Figure 1). In 2011, at the time of the nuclear accident, the village had a population of ~1,500 individuals, and following the radioactive incident, all residents were forced to evacuate. In June 2016, the radiation levels declined, and evacuation orders were lifted for most parts of the village. Seven years have passed since the residents could return, and in November 2023, 464 people were living in the village. However, more than 60% of the village's registered population still have not returned.

Following the nuclear accident, obesity has increased among registered residents of Katsurao Village. Nevertheless, no studies focusing on the evacuation of residents after the nuclear accident in Chornobyl and the subsequent increase in obesity have been found. However, obesity is related to various diseases, including type 2 diabetes (12), and places a considerable burden on healthcare expenditure (13). Therefore, from a public health perspective, it is important to understand the factors underlying the obesity trends observed in this region and implement measures to prevent obesity.

This study aimed to determine the prevalence of obesity among residents of Katsurao Village, Fukushima Prefecture, after the evacuation order was lifted in 2016 and compare the prevalence of obesity by place of residence (inside or outside the village) (14).

## 2 Materials and methods

### 2.1 Participants

Data from individuals aged 40–74 years in Katsurao Village who underwent health checkups, between 2016 and 2023 were used in the analyses. Health checkups were conducted in each municipality for people enrolled in the national health insurance (15). In Katsurao Village, ~150 people receive annual checkups. The results of these checkups are considered indicators of community health status in Japan (16). An advantage of this collected data is that it is comparable with datasets from other municipalities. This study was conducted in accordance with the Declaration of Helsinki and approved by the Fukushima Medical University Ethics Committee (approval no: #REC2023-117; date: 04 September 2023).

### 2.2 Data collection

The registered population of Katsurao village from 2016 to 2023 was determined using the monthly population data published in the public relations magazine *Katsurao*. From the medical examinations performed between 2016 and 2023, the following was obtained: number of examinees, age, sex, place of residence (inside or outside the village), and body mass index (BMI). Lifestyle habits, including smoking habit (yes, no), exercise for more than 30 min a day at least twice a week (yes, no), eating speed compared to others (fast, normal, slow), and alcohol consumption (non-drinker, < 6 oz, 6–12 oz, 12–18 oz, more than 18 oz), were extracted from the health checkup questionnaire (see Supplementary Table 1).

### 2.3 Standard BMI value

According to World Health Organization international standards, a BMI ≥ 30 kg/m^2^ corresponds to obesity. However, Asians, including Japanese, are at high risk of developing type 2 diabetes and cardiovascular disease, even with a BMI of < 30 kg/m^2^. Therefore, in Japan, an individual with a BMI of 25 kg/m^2^ or above is considered obese (17).

### 2.4 Data analyses

Based on analysis of the collected data, we determined the following:

- The population trend in Katsurao Village from 2016 to 2023, that is, the number of people by place of residence (inside and outside the village).
- The number of people who underwent health checkups each year from 2016 to 2023 by place of residence (inside and outside the village).
- The percentage of people with obesity (BMI ≥ 25 kg/m^2^) from 2016 to 2023 by place of residence (inside and outside the village).
- For the 2023 data, we conducted a multiple logistic regression analysis using the presence or absence of obesity as the objective variable, and sex, age, place of residence, exercise habits, smoking habits, drinking habits, and eating speed were explanatory variables.
- We conducted a multiple logistic regression analysis using data from individuals examined outside the village in 2023. The objective variable was the presence of obesity, and the explanatory variables included age, sex, place of residence, exercise habits, smoking habits, drinking habits, and eating speed.
- Confidence intervals (CIs), *p*-values, and odds ratios (ORs) were calculated. Data analyses were performed using IBM SPSS Statistics version 28.0.1.0 (IBM Corp., Armonk, NY, USA) (142) with a two-sided test set at a significance level of 5%.

## 3 Results

### 3.1 Temporal trend for number of residents in Katsurao Village

**Figure 2:**
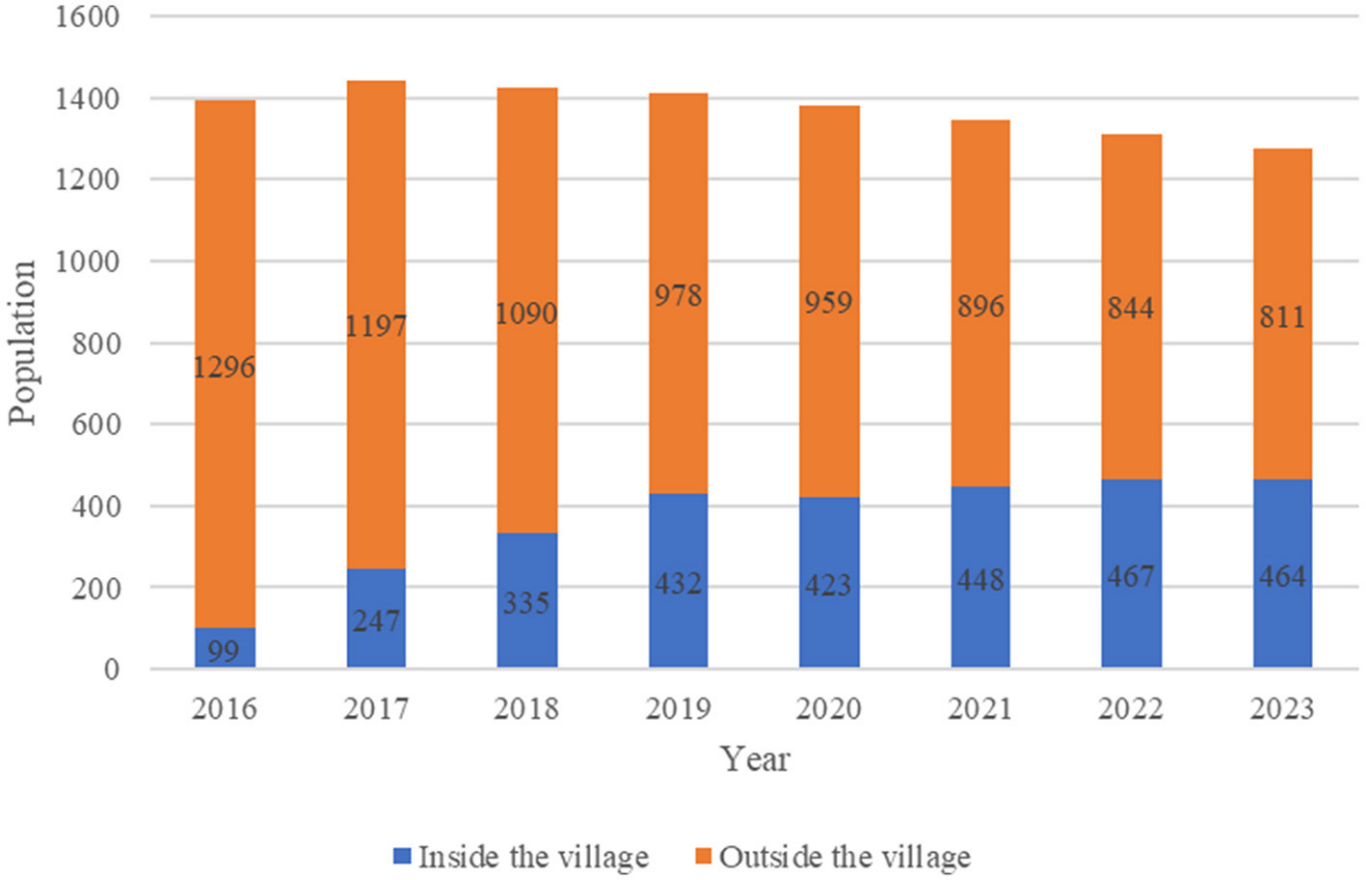
Population trend of Katsurao Village after the evacuation order was lifted.

The temporal trend of the population of Katsurao Village by place of residence (inside and outside the village) after 2016, when the evacuation order was lifted, is shown in Figure 2. As of 1 November 2016, the registered population of Katsurao Village was 1,395, with 99 (7.1%) residents in the village. This was followed by 1,444 [247 (17.1%)] in 2017, 1,425 [335 (23.5%)] in 2018, 1,410 [432 (30.6%)] in 2019, 1,382 [423 (30.6%)] in 2020, 1,344 [448 (33.3%)] in 2021, 1,311 [467 (35.6%)] in 2022, and 1,275 [464 (36.4%)] in 2023. Although the number of people returning to the village increased for the first few years, not much has changed since then.

### 3.2 Changes in the number of people who had been receiving health checkups in Katsurao Village

**Figure 3:**
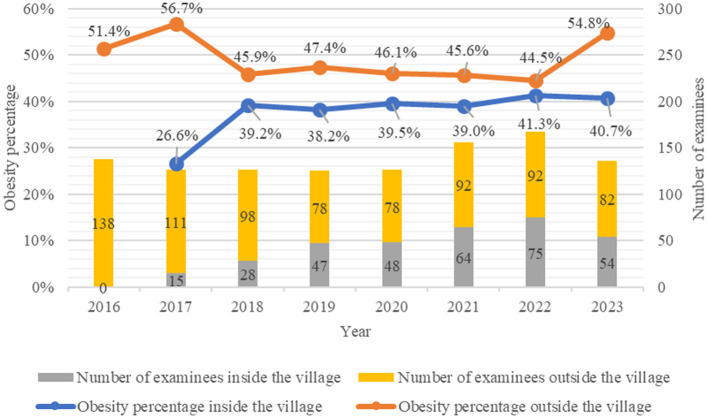
Number of people who underwent health checkups and the obesity rate by place of residence (inside and outside the village) after the evacuation order was lifted.

The number of residents who had been receiving health checkups by place of residence (inside and outside the village) after 2016 is presented in Figure 3. In 2016, when the evacuation order was lifted, 138 people received health checkups, and no examinees were present in the village. This was followed by 126 [15 (11.9%)] in 2017, 126 [28 (22.2%)] in 2018, 125 [47 (37.6%)] in 2019, 126 [48, (38.1%)] in 2020, 156 [64 (41.0%)] in 2021, 167 [75 (44.9%)] in 2022, and 136 [54 (39.7%)] in 2023.

### 3.3 Trends in the percentage of people with a BMI ≥ 25 kg/m^2^ living inside and outside of Katsurao Village

The temporal trends in the percentage of people with a BMI ≥ 25 kg/m^2^ by place of residence (inside and outside the village) since 2016 are shown in the line graphs of Figure 3. Among the residents living outside the village, the percentage of people with a BMI ≥ 25 kg/m^2^ was 51.4% (71 people) in 2016, 56.7% (63) in 2017, 45.9% (45) in 2018, 47.4% (37) in 2019, 46.1% (36) in 2020, 45.6% (42) in 2021, 44.5% (41) in 2022, and 54.8% (45) in 2023. Among residents living in the village, the percentage was 26.6% (4) in 2017, 39.2% (11) in 2018, 38.2% (18) in 2019, 39.5% (19) in 2020, 39.0% (25) in 2021, 41.3% (31) in 2022, and 40.7% (22) in 2023. The percentage of people with a BMI ≥ 25 kg/m^2^ has consistently been higher among the residents living outside the village compared to those living inside the village.

### 3.4 Results of the multiple logistic regression analysis with obesity as the outcome of interest

The results of the multiple logistic regression analysis using place of residence (inside or outside the village) data from 2023 with obesity as the outcome of interest are shown in Table 1. Obesity was significantly higher in those living outside the village (OR: 2.273; CI: 1.061–4.866; *p* = 0.035).

**Table 1:** Results of the multiple logistic regression analysis with obesity as the outcome variable (2023).

**Factors**	**Class**	**Obesity rate (%)**	**Unadjusted OR**	**95%CI**	***p*-value**	**Adjusted OR**	**95%CI**	***p*-value**
Age	Under 64 years old	44.7%	1.00			1.00		
	Over 65 years old	51.7%	1.324	0.651–2.693	0.438	1.383	0.636–3.011	0.414
Sex	Male	50.7%	1.00			1.00		
	Female	47.7%	1.128	0.575–2.212	0.726	0.795	0.352–1.798	0.582
Place of residence	Inside the village	40.7%	1.00			1.00		
	Outside the village	54.9%	1.769	0.882–3.547	0.108	2.273	1.061–4.866	0.035
Exercise habits	Yes	46.8%	1.00			1.00		
	No	50.6%	1.636	0.691–3.872	0.263	0.997	0.458–2.172	0.994
Drinking habits	Non-drinker	45.1%	1.00			1.00		
	< 6 oz	54.5%	1.462	0.638–3.352	0.369	1.953	0.779–4.900	0.154
	6–12 oz	47.8%	1.117	0.435–2.867	0.818	1.119	0.387–3.232	0.836
	12–18 oz	66.7%	2.437	0.419–14.176	0.321	3.026	0.459–19.947	0.250
	More than 18 oz	66.7%	2.437	0.211–28.121	0.475	3.016	0.232–39.197	0.399
Eating speed	Slow	60.0%	1.00			1.00		
	Rapid	53.6%	0.767	0.330–1.779	0.536	0.675	0.277–1.642	0.386
	Moderate	46.9%	1.300	0.300–5.637	0.726	1.565	0.328–7.463	0.574
Smoking habits	Non-smoker	50.0%	1.00			1.00		
	Current smoker	45.5%	0.833	0.333–2.083	0.696	0.654	0.221–1.937	0.443

### 3.5 Results of multiple logistic regression analysis focusing on residents outside the village with obesity as an outcome

Table 2 shows the results of the multiple logistic regression analysis with obesity as the outcome, based on data from residents outside the village in 2023. No questionnaire items were found to be significantly related to the presence of obesity.

**Table 2:** Results of the multiple logistic regression analysis with obesity as the outcome variable among residents outside the village (2023).

**Factors**	**Class**	**Obesity rate (%)**	**Unadjusted OR**	**95%CI**	***p*-value**	**Adjusted OR**	**95%CI**	***p*-value**
Age	Under 64 years old	46.4%	1.00			1.00		
	Over 65 years old	59.3%	1.678	0.669–4.211	0.27	2.095	0.71–6.182	0.18
Sex	Male	55.0%	1.00			1.00		
	Female	54.8%	0.99	0.415–2.365	0.983	0.963	0.294–3.155	0.95
Exercise habits	Yes	58.6%	1.00			1.00		
	No	52.8%	0.791	0.317–1.974	0.615	0.727	0.257–2.055	0.547
Drinking habits	Non-drinker	52.1%	1.00			1.00		
	< 6 oz	64.3%	1.656	0.483–5.672	0.422	1.931	0.499–7.473	0.34
	6–12 oz	53.3%	1.051	0.329–3.36	0.933	1.113	0.278–4.46	0.88
	12–18 oz	50.0%	0.92	0.12–7.076	0.936	1.17	0.134–10.241	0.887
	More than 18 oz	100.0%	1.49E+09		1	1.11E+09		1
Eating speed	Slow	100.0%	1.00			1.00		
	Rapid	50.0%	1.1	0.345–3.504	0.872	1.164	0.344–3.943	0.807
	Moderate	52.4%	1.62E+09		0.999	2.41E+09		0.999
Smoking habits	Non-smoker	55.9%	1.00			1.00		
	Current smoker	50.0%	0.789	0.25–2.498	0.688	1.185	0.283–4.962	0.816

## 4 Discussion

Here, we reported the effects of long-term evacuation following a radioactive disaster on the development of health risks in the affected population over a long period. The effects of long-term relocation on health after natural disasters (18) have been previously demonstrated after radiation disasters (19).

Relocation and living outside the village were significantly associated with obesity. Generally, various factors contributed to the increase in BMI in this population. Although not significant from the items in this study, changes in the living environment and new lifestyle habits, such as in eating and exercise (20, 21), lead to an increase in BMI. From our observations of the living conditions of our residents, the lower BMI in residents who returned to the village may be attributed to a resumption of their daily lives eating and exercise habits. Typically, the individuals in the village engage in agricultural activities.

Obesity should be considered among the factors leading to the rapid increase in the number of people certified for long-term care in formerly evacuated areas following the nuclear accident, including Katsurao Village (22). A significant association exist between obesity and frailty (23). Radioactive-affected areas are experiencing population displacement and an aging population (24), making long-term care an urgent issue (25). Interventions to prevent obesity in individuals from the affected areas could address the social challenges of long-term care in this region.

It is important to consider obesity as a long-term health problem following a radiation disaster. The obesity rate has consistently been higher among people living outside the village. Losing weight for individuals with obesity requires time and effort, and exercises are more challenging to complete if an individual is obese. Conventional support for residents should be based on their actual living conditions. However, when nearly 70% of registered residents live outside the village, it is difficult for the municipal office to take the lead in continuing resident support (26). Our results suggest that sharing information with health institutions and health-related organizations in evacuation destinations and providing early intervention to address anticipated health risks are necessary.

This study has some limitations. First, the data collected was from a small village, and the sample size was small. Second, this study examined only BMI data. Further research is needed to determine other health-related factors among affected individuals in the village and surrounding areas affected by evacuation. Third, the medical examination item “place of residence” may not accurately reflect the evacuee/returnee status. After more than 10 years since the nuclear accident, it may no longer be possible to categorize those living outside the village as “evacuees” and those living within the village as “returnees.” Therefore, in the future, there will be a need for interventions to address health issues that consider the inflow and outflow of populations.

This study tracked the health status of residents over a long period until 2023, 12 years after the nuclear accident. The health effects of long-term evacuation on residents can be demonstrated as the risk factors for obesity, specifically BMI. Obesity is a health issue faced by residents transitioning to new lifestyles, and countermeasures are indispensable. When implementing community health promotion after a disaster, it is necessary to consider interventions immediately after the disaster occurs.

## Data availability statement

The original contributions presented in the study are included in the article/Supplementary material, further inquiries can be directed to the corresponding author.

## Author contributions

NI: Writing – original draft, Writing – review & editing. MTa: Conceptualization, Writing – review & editing. NM: Conceptualization, Methodology, Writing – review & editing. IA: Conceptualization, Data curation, Methodology, Writing – review & editing. AF: Conceptualization, Investigation, Writing – review & editing. HS: Investigation, Methodology, Writing – review & editing. TA: Conceptualization, Investigation, Writing – review & editing. CY: Conceptualization, Investigation, Writing – review & editing. MS: Conceptualization, Writing – review & editing. TZ: Data curation, Writing – review & editing. MTs: Supervision, Writing – review & editing.
